# Valve-In-Valve TAVI in a Patient With Dextrocardia, Situs Inversus, and Challenging Commissural Alignment

**DOI:** 10.1016/j.jaccas.2025.105833

**Published:** 2025-10-25

**Authors:** Ho-On Alston Conrad Chiu, Ka-Chun Un, Chun-Ka Wong, Shu-Yue Sze, Ching-Wei Lee, Frankie Chor-Cheung Tam, Kwong-Yue Eric Chan, Daniel Tai-Leung Chan, Gilbert H.L. Tang, Simon Cheung-Chi Lam

**Affiliations:** a Cardiology Division, Department of Medicine, Li Ka Shing Faculty of Medicine, The University of Hong Kong, Hong Kong, China; b Cardiology Division, Department of Medicine, Queen Mary Hospital, Hong Kong, China; c Division of Cardiology, Department of Internal Medicine, Taipei Veterans General Hospital, Taipei, Taiwan; d Cardiac Medical Unit, Grantham Hospital, Hong Kong, China; e Department of Cardiothoracic Surgery, Queen Mary Hospital, Hong Kong, China; f Department of Surgery, Li Ka Shing Faculty of Medicine, The University of Hong Kong, Hong Kong, China; g Department of Cardiovascular Surgery, Mount Sinai Health System, New York, New York, USA

**Keywords:** dextrocardia, transcatheter aortic valve implantation, valve-in-valve

## Abstract

Valve-in-valve transcatheter aortic valve implantation (ViV-TAVI) for dextrocardia is challenging. An 81-year-old man with dextrocardia and situs inversus developed prosthetic aortic stenosis. Loading Evolut FX+ C-tab 180° upside down in a delivery catheter facilitates commissural alignment during ViV-TAVI in dextrocardia. To accommodate anatomical variations, the flush-port position can be adjusted. In this case, the flush port was positioned 5-o’clock (60° from the usual 3-o’clock position) to align the TAVI valve with former prosthesis, as the former prosthetic valve commissure was oriented 60° from native commissures to preserve coronary access for abnormal left coronary artery originating from native commissures. This case illustrates strategies for commissural alignment during ViV-TAVI in dextrocardia.

## Graphical Abstract

**Figure:**
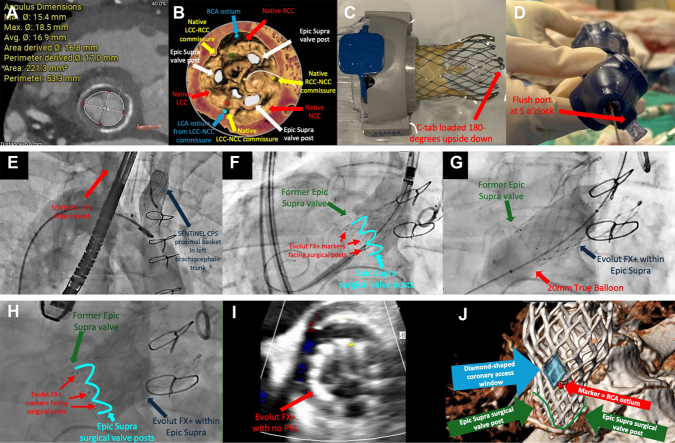

Achieving optimal commissural alignment during valve-in-valve transcatheter aortic valve implantation (ViV-TAVI) in patients with dextrocardia is especially challenging and has been rarely described. We present a case demonstrating practical alignment strategies.

An 81-year-old man with dextrocardia and situs inversus, who underwent surgical aortic valve (SAV) replacement with a 21-mm Epic Supra valve (Abbott Structural Heart) in 2013, developed severe prosthetic stenosis. The heart team deemed him at high risk for redo SAV replacement.

Computed tomography analysis demonstrated feasible anatomy for transfemoral ViV-TAVI. The SAV had a true inner diameter of 17 mm, an area of 221.3 mm^2^, and a perimeter of 53.3 mm (Figure 1A). Thus, a 23-mm Evolut FX+ valve (Medtronic), a new-generation supra-annular transcatheter heart valve (THV) with a dedicated commissural alignment implantation algorithm and enlarged diamond-shaped windows facilitating coronary access, was chosen. However, abnormal left coronary artery ostia originating from the native left coronary cusp and noncoronary cusp commissure posed difficulty in achieving commissural alignment. To preserve coronary accesses, the SAV was oriented in an unconventional manner, with the SAV commissure positioned at approximately 60° from native commissures (Figure 1B).

**Figure 1:**
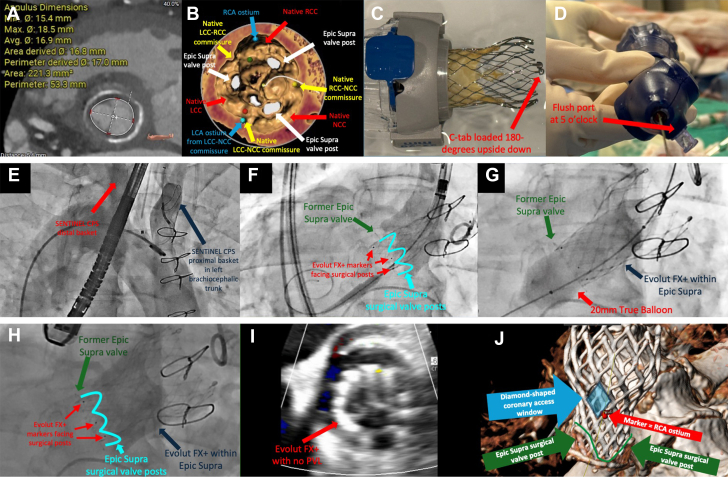
ViV-TAVI in Dextrocardia, Situs Inversus, and Challenging Commissural Alignment (A) Annular measurements. (B) Left coronary artery (cyan) originating from the LCC-NCC commissure. The SAV was not aligned with native commissures. (C) C-tab loaded 180° upside down. (D) Flush-port position at 5-o’clock. (E) SENTINEL CPS deployed via the left radial artery. (F to H) Deployment, postdilatation, and final position of the Evolut FX+ valve. (I) No postoperative paravalvular leak. (J) Postoperative CT showing alignment between the diamond-shaped coronary access window and the former SAV. CPS = Cerebral Protection System; CT = computed tomography; LCA = left coronary artery; LCC = left coronary cusp; NCC = noncoronary cusp; PVL = paravalvular leak; RCA = right coronary artery; RCC = right coronary cusp; SAV = surgical aortic valve; TAVI = transcatheter aortic valve implantation; ViV = valve-in-valve.

It was determined that ideal THV deployment should follow alignment of the SAV. To achieve commissural alignment in the setting of dextrocardia, C-tab was first loaded 180° upside down in the delivery catheter to mirror the usual orientation (Figure 1C). After the alignment of the SAV, the flush port was rotated 60° from the usual 3-o’clock position and ultimately positioned at 5-o’clock (Figure 1D). The SENTINEL Cerebral Protection System (Boston Scientific) was deployed via the left radial artery in a reversed configuration (Figure 1E). After determining optimal THV positioning fluoroscopically, the THV was successfully deployed after the alignment of the SAV, with subsequent postdilatation using a 20-mm True balloon (Becton Dickinson) (Figures 1F to 1H). The postoperative transvalvular gradient was 26/14 mm Hg, with no paravalvular leak or aortic regurgitation (Figure 1I). Post-TAVI computed tomography demonstrated commissural alignment with the former SAV, consistent with fluoroscopic findings (Figure 1J). At 30 days, the patient's condition improved to NYHA functional class I with no paravalvular leak and a transvalvular gradient of 29/15 mm Hg.

This case demonstrated practical strategies to achieve commissural alignment for ViV-TAVI in dextrocardia and situs inversus.

## Funding Support and Author Disclosures

Dr Tang has received speaker honoraria from and served as a physician proctor, consultant, advisory board member, TAVR publications committee member, RESTORE study steering committee member, APOLLO trial screening committee member, and IMPACT MR steering committee member for Medtronic; has received speaker honoraria from and served as a physician proctor, consultant, advisory board member, and TRILUMINATE trial anatomic eligibility and publications committee member for Abbott Structural Heart; has served as an advisory board member for Boston Scientific, a consultant and physician screening committee member for Shockwave Medical, and a consultant for Anteris Technologies, Philips, Edwards Lifesciences, Peijia Medical, and Shenqi Medical Technology; and has received speaker honoraria from Siemens Healthineers. All other authors have reported that they have no relationships relevant to the contents of this paper to disclose.

